# Type 1 diabetes recurrence after SARS‐CoV‐2 infection in a subject with pancreas transplantation

**DOI:** 10.1002/edm2.364

**Published:** 2022-10-28

**Authors:** Valentina Popolla, Alessandro Rizzi, Linda Tartaglione, Alfredo Pontecorvi, Dario Pitocco

**Affiliations:** ^1^ Department of Internal Medicine Fondazione Policlinico Universitario Agostino Gemelli IRCCS Rome Italy; ^2^ Diabetes Care Unit Fondazione Policlinico Universitario Agostino Gemelli IRCCS Rome Italy; ^3^ Department of Endocrinology Fondazione Policlinico Universitario Agostino Gemelli IRCCS Rome Italy

## Abstract

**Background:**

During COVID‐19 pandemic, several studies have demonstrated a strong link between SARS‐CoV‐2 infection and diabetes mellitus. Hyperglycaemia is a frequent event during the infection, also in patients without a history of diabetes. Furthermore, several cases of diabetic ketoacidosis during COVID‐19 disease have been described. No data are available about the effects of SARS‐CoV‐2 infection on glycaemic control in pancreas transplant patients.

**Case presentation:**

A 45‐year‐old woman affected by type 1 diabetes mellitus was treated with kidney‐pancreas transplantation in 2015, 6 years before COVID‐19 infection. After transplantation, insulin therapy was stopped with a good glycaemic control during the following years.After SARS‐CoV‐2 infection, she developed severe hyperglycaemia requiring insulin therapy again. During the acute phase of the infection, the detection of antibodies against islet cells (ICA) and against glutamic acid decarboxylase (GAD) was found positive.

**Conclusions:**

The onset of hyperglycaemia after SARS‐CoV‐2 infection might be the result of a direct virus‐induced toxicity or the effect of a virus‐mediated activation of autoimmunity.

## Graphical abstract

During COVID‐19 pandemic, several studies have demonstrated a strong link between SARS‐CoV2 infection and diabetes mellitus. Little is known about the effects of SARS‐CoV‐2 infection on glycaemic control in pancreas transplant patients.

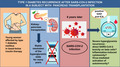

## 1 CASE PRESENTATION

Type 1 diabetes (T1DM) is a metabolic disorder caused by an autoimmune process that damages pancreatic β‐cells. The serological markers of T1DM are represented by antibodies against islet cells (ICA), glutamic acid decarboxylase (GAD), tyrosine phosphatase (IA2), zinc‐transporter 8 (ZnT8) and insulin (IAA).

Pancreas transplantation is a therapeutic option for subjects with type 1 diabetes with severe glycaemic variability and/or rapid evolution of chronic complications.

After pancreas transplantation, insulin therapy can be discontinued.

With the emergence of the pandemic COVID‐19, a strong bidirectional link between diabetes and SARS‐CoV‐2 infection has been demonstrated.

In particular, an interesting association between COVID‐19 and type 1 diabetes has emerged.

A case of acute diabetic ketoacidosis (DKA) during SARS‐CoV‐2 infection has been described, in absence of serum autoantibodies against islet cells.^1^

Furthermore, a marked increase in acute diabetic ketoacidosis (DKA) has been reported among German children and adolescents infected by SARS‐CoV2 2,^2^ suggesting a relationship between COVID‐19 and new onset of type 1 diabetes.

No data are available about the effects of SARS‐CoV‐2 infection on glycaemic control in pancreas transplanted patients.

We report the case of a patient affected by type 1 diabetes mellitus treated with kidney‐pancreas transplantation, who developed acute severe hyperglycaemic state after SARS‐CoV‐2 infection with requirement of insulin therapy again, in the presence of detectable serum anti‐glutamic acid decarboxylase (GAD) antibodies and anti‐islet cell antibodies (ICA).

The patient, a 45‐year‐old woman affected by type 1 diabetes mellitus complicated by chronic kidney disease, Charcot arthropathy and proliferative diabetic retinopathy, underwent kidney‐pancreas transplantation in 2015 and started immunosuppressive therapy based on tacrolimus, mycophenolate ad low doses of corticosteroids.

Before transplantation, she was on basal‐bolus insulin therapy. The blood glucose levels stably exceeded the normal range, with frequent episodes of hyperglycaemia above 500 mg/dl. The glycated haemoglobin (HbA1c) values were regularly indicative of a poor blood glucose control status. The renal function was also severely impaired and the patient performed dialysis for many years before transplantation.

After kidney–pancreas transplantation, dialysis and insulin therapy were stopped.

In the following months and years, the blood glucose levels and HbA1c values were stably in the normal range.

In April 2021, the patient developed SARS‐CoV‐2 infection with COVID‐related interstitial pneumonia.

During the acute phase of the infection, as recommended by guidelines, prednisone was replaced by dexamethasone 6 mg per day.

On 19 April, the patient was moved to the Intensive care unit where she initially underwent endotracheal intubation and later tracheotomy.

During the Intensive care unit monitoring, dexamethasone 6 mg per day was continued. As regards the immunosuppressive therapy, mycophenolate and tacrolimus were discontinued only for a few days.

Despite the administration of high doses of corticosteroids, during this phase, the patient showed a good glycaemic control without the necessity of insulin therapy.

In June 2021, in the light of substantial clinical improvement, dexamethasone 6 mg was replaced by prednisone 10 mg per day.

In July 2021, the patient was tested negative for SARS‐CoV‐2 and was moved to a rehabilitation ward. During rehabilitation, the blood glucose levels were permanently normal (values between 70 and 100 mg/dl).

On 27 July, the patient was tested positive for SARS‐CoV‐2 again. She was discharged at home euglycaemic, in good clinical conditions.

In August 2021, because of the occurrence of fever and dyspnoea, she was admitted again. During this last hospitalization, she developed severe acute hyperglycaemia (glucose blood levels >1000 mg/dl), and insulin therapy was started. The detection of anti‐glutamic acid decarboxylase antibodies (GAD) and anti‐islet cell antibodies (ICA) was found positive. C‐peptide levels were lower than normal. The pancreatic exocrine function was also tested, with evidence of normal amylase and lipase blood levels.

Because of the patient's poor clinical conditions, the transplanted pancreas biopsy could not be performed.

The patient was discharged on basal‐bolus insulin therapy. During the following months, she maintained a chronic state of hyperglycaemia requiring increasing doses of insulin.

Several studies have demonstrated a strong linkage between COVID‐19 and diabetes.

The reason might first be found in the mechanisms of the virus entry into cells.

Multiple receptors are involved in SARS‐CoV‐2 uptake. In particular, an essential role is played by angiotensin‐converting enzyme 2 (ACE2), a transmembrane glycoprotein with proteolytic activity found on the surface of many cell types. ACE2 has also been found in human pancreatic islets, with a preferential expression in the insulin‐producing pancreatic β‐cells, in pancreas microvasculature pericytes and in rare scattered ductal cells.

This evidence suggests that SARS‐CoV‐2 might potentially cause diabetes through a direct destruction of beta‐cells or through the infection of pancreatic microvasculature and/or ductal cells thus impairing insulin secretion.^3^

Another possible hypothesis is that of the immune‐mediated damage.

It has been observed that, in genetically predisposed individuals, hyperactivation of the immune response against SARS‐CoV‐2 virus may result in the development of autoimmune disorders. Several cases of antiphospholipid antibody syndrome, Kawasaki Syndrome, Guillain‐Barré Syndrome, Miller Fisher Syndrome, idiopathic thrombocytopenic purpura and autoimmune haemolytic anaemia have been described.^4^

It has been hypothesized that SARS‐CoV‐2 could represent a potential trigger for autoimmunity, through not well‐defined mechanisms.

As regards diabetes and the onset of acute hyperglycaemia, we may suppose that SARS‐CoV‐2 infection could induce pathological immune reactions against key antigens in pancreatic beta‐cells.

One possible pathogenetic mechanism could be the activation of autoimmune processes as consequence of a dysregulation in the cytokines production. Different studies have shown that COVID‐19 infection determines an increase in the general inflammation markers, a higher production of inflammatory cytokines and lowered levels of lymphocytes in the blood.^5^

Among cytokines, a key role is played by Interleukin‐6 (IL‐6), a pleiotropic molecule involved in the early stages of the inflammatory response.

It has been demonstrated that during SARS‐CoV‐2 infection, higher levels of IL‐6 are produced.

Among its multiple functions, IL‐6 controls the secretion of antibodies from B cells and the activation, differentiation and survival of T cells. If dysregulated, this process could lead to autoimmune disorders.

Also, endothelitis is described as a typical sign of SARS‐CoV‐2 infection in various organs. The presence of ACE2 in pancreatic pericytes could lead to local inflammation and hyperactivation of immune responses.

Hyperinflammation and imbalanced immune reactions, in certain conditions, could cause the development of autoimmune diseases.

In the present case, SARS‐CoV‐2 infection has determined severe hyperglycaemia with the requirement of insulin therapy again in a pancreas transplanted subject who had a good metabolic control before COVID‐19 disease.

Furthermore, at the onset of hyperglycaemia, the detection of antibodies against islet cells and glutamic acid decarboxylase was performed and was found positive.

Type 1 diabetes is considered a T‐cell‐mediated autoimmune pathology. The role of autoantibodies in the pathogenesis of the disease is not clear. It has been supposed that autoantibodies are not directly involved in the destruction of β‐cells but more likely reflect the presence of an autoimmune activity, thus representing the serological markers of the disease.

After transplantation, a long‐term persistence of humoral autoimmunity can be observed, generally without any impact on the patient's glycaemic control.

In our case, we can hypothesize a SARS‐CoV‐2‐induced exacerbation of autoimmunity but the role of positive ICA and GAD autoantibodies in this process is unknown. A measurement of specific type 1 diabetes autoantibodies performed between 2015 and 2021 (after pancreas transplantation and before SARS‐CoV‐2 infection) could have helped us to give a more precise explanation of the observed phenomenon.

In conclusion, the infection might have induced a damage in the functional reserve of the transplanted pancreas through a direct SARS‐CoV‐2 toxicity on beta‐cells and/or through a direct inflammation‐induced cytotoxicity and/or through the activation of autoimmune processes as consequence of the inflammatory status.

We should note that hyperglycaemia occurred after the second infection, which was clinically milder than the first.

This might seem to disagree with the hypothesis of a direct role of the infection in the development of hyperglycaemia.

However, the second infection was diagnosed only a few weeks after the first.

It is possible that the second infection so soon after the first was relevant to the loss of glucose control.

We should also take into account that, because of SARS‐CoV‐2 infection, the immunosuppressive therapy was temporarily stopped. As a matter of fact, as recommended by guidelines, during the acute phase of the infection, the dosage of corticosteroids was increased while mycophenolate and tacrolimus were temporarily discontinued.

Nevertheless, this discontinuation lasted only a few days, so its contribute to the development of hyperglycaemia, as a result of the organ rejection, could not be reasonable. Furthermore, during the interruption of immunosuppressive therapy, despite the concomitant administration of high corticosteroids doses, the patient was stably euglycaemic.

The study of alloreactivity was not performed. The transplanted pancreas biopsy could have been a tool to study the histological aspect of the organ, thus consenting to answer our doubts but, because of the patient's poor clinical conditions, the procedure could not be performed.

Further studies should be designed to give an answer to the observations described in this clinical case.

## AUTHOR CONTRIBUTIONS

**Valentina Popolla:** Data curation (supporting); investigation (equal); writing – original draft (lead); writing – review and editing (lead). **Alessandro Rizzi:** Data curation (equal); visualization (supporting); writing – original draft (supporting). **Linda Tartaglione:** Data curation (equal); visualization (supporting). **Alfredo Pontecorvi:** Conceptualization (supporting); visualization (supporting). **Dario Pitocco:** Conceptualization (lead); supervision (lead); validation (lead).

## FUNDING INFORMATION

None.

## CONFLICT OF INTEREST

Authors declare no conflicts of interest.

## CONSENT FOR PUBLICATION

Written informed consent was obtained from the patient for publication of this case report. A copy of the written consent is available for the Editor of this journal.

## Data Availability

data can be found in the patient’s medical records
